# Neural representation of the sensorimotor speech–action-repository

**DOI:** 10.3389/fnhum.2013.00121

**Published:** 2013-04-04

**Authors:** Cornelia Eckers, Bernd J. Kröger, Katharina Sass, Stefan Heim

**Affiliations:** ^1^Department of Phoniatrics, Pedaudiology, and Communication Disorders, Medical School, RWTH Aachen UniversityAachen, Germany; ^2^Cognitive Computation and Applications Laboratory, School of Computer Science and Technology, Tianjin UniversityTianjin, Peoples' Republic of China; ^3^Department of Psychiatry, Psychotherapy and Psychosomatics, Medical School, RWTH Aachen UniversityAachen, Germany; ^4^JARA – Translational Brain MedicineJülich and Aachen, Germany; ^5^School of Psychology, University of QueenslandBrisbane, QLD, Australia; ^6^Department of Psychiatry, Psychotherapy, and Psychosomatics, Section Structural Functional Brain Mapping, Medical School, RWTH Aachen UniversityAachen, Germany; ^7^Research Centre Jülich, Institute of Neuroscience and Medicine (INM-1)Jülich, Germany; ^8^Department of Neurology, Section Clinical and Cognitive Neurosciences, Medical School, RWTH Aachen UniversityAachen, Germany

**Keywords:** mental syllabary, supramodal, sensorimotor, motor theory, syllable processing, speech–action-repository, fMRI, conjunction analysis

## Abstract

A speech–action-repository (SAR) or “mental syllabary” has been proposed as a central module for sensorimotor processing of syllables. In this approach, syllables occurring frequently within language are assumed to be stored as holistic sensorimotor patterns, while non-frequent syllables need to be assembled from sub-syllabic units. Thus, frequent syllables are processed efficiently and quickly during production or perception by a direct activation of their sensorimotor patterns. Whereas several behavioral psycholinguistic studies provided evidence in support of the existence of a syllabary, fMRI studies have failed to demonstrate its neural reality. In the present fMRI study a reaction paradigm using homogeneous vs. heterogeneous syllable blocks are used during overt vs. covert speech production and auditory vs. visual presentation modes. Two complementary data analyses were performed: (1) in a logical conjunction, activation for syllable processing independent of input modality and response mode was assessed, in order to support the assumption of existence of a supramodal hub within a SAR. (2) In addition priming effects in the BOLD response in homogeneous vs. heterogeneous blocks were measured in order to identify brain regions, which indicate reduced activity during multiple production/perception repetitions of a specific syllable in order to determine state maps. Auditory-visual conjunction analysis revealed an activation network comprising bilateral precentral gyrus (PrCG) and left inferior frontal gyrus (IFG) (area 44). These results are compatible with the notion of a supramodal hub within the SAR. The main effect of homogeneity priming revealed an activation pattern of areas within frontal, temporal, and parietal lobe. These findings are taken to represent sensorimotor state maps of the SAR. In conclusion, the present study provided preliminary evidence for a SAR.

## Introduction

Crompton (1982) was the first who mentioned storage for articulatory routines of syllables in the context of explaining different speech errors. This notion was further developed by Levelt (1989, 1992, 1993) and subsequently by Levelt and Wheeldon (1994). They postulated a model of speech production comprising two different storages. A mental lexicon is assumed as storage for concepts, lemmas, and phonological representations; a mental syllabary is assumed as storage for motor plans (gesture scores, see also Levelt et al., 1999 and Levelt, 2001). While the assumption of a mental lexicon is widely accepted (e.g., Levelt, 1989; Dell et al., 1993; Elman, 2004) the assumption of a mental syllabary, based on reaction time experiments (Levelt and Wheeldon, 1994), is still being under debate (Aichert and Ziegler, 2004).

The concept of a syllabary implies that a speaker does not need to assemble a frequent syllable each time online from subsyllabic units but simply activates the gesture score of a syllable, which results in a more efficient and faster production (Levelt and Wheeldon, 1994). Thus, a syllabary would be an efficient instrument of conserving neuronal processing time by retrieval of stored neuronal syllabic patterns. Further arguments for the existence of a mental syllabary were provided by Cholin et al. (2006). They determined a syllable frequency effect in monosyllabic and bisyllabic pseudowords in which the first syllable bore the frequency manipulation.

Moreover, neuroimaging studies were conducted in order to identify neuroanatomical correlates of a mental syllabary (cf. Riecker et al., 2008; Brendel et al., 2011). In Riecker et al. (2008) subjects were asked to read aloud visually presented bisyllabic pseudowords during functional magnetic resonance imaging (fMRI). They found main effects of speech production comprising cortical parts of frontal, temporal, and parietal as well as subcortical areas. A significant effect of syllable frequency did not emerge. Brendel et al. (2011) investigated the influence of syllable frequency on speech motor control processes, i.e., overt reading of pseudowords as well. They found a speech production network which is common to high-frequent simple syllables (i.e., consonant (C)-vowel (V) combinations, e.g., [ba:] or [be:]), high-frequent complex syllables (i.e., CCV combinations, e.g. [bli:] or [blu:]), low-frequent simple, and low-frequent complex syllables including cortical frontal, temporal, and parietal as well as subcortical areas. Focused on the mental syllabary, the reaction time analysis showed a frequency effect but in contrast, fMRI data revealed no effect of syllable frequency. In summary, experimental phonetic studies to prove the existence of the mental syllabary are rare and their results are ambivalent (Benner et al., 2007).

**Figure 1:**
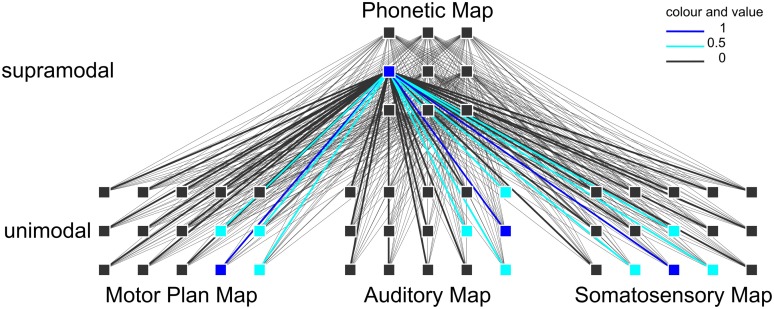
**Example of a neuronal self-organizing network and a specific syllable activation.** Activation within the self-organizing phonetic map leads to activation of every neuron within the state maps (motor map, auditory map, somatosensory map) by interconnection of these neurons. By different link weights some neurons are fully activated (dark blue) and some are weakly activated (light blue) and others are zero-activated (bold black).

However, these imaging studies were limited to the investigation of syllable processing only during speech production and they looked for only one specific region, which hosts the syllabary. In the theoretical computer-implemented neurofunctional speech model of Kröger et al. (2009, 2011) the close relationship of speech production and speech perception is postulated as mentioned by Liberman et al. (1967), Liberman and Mattingly (1985), or Fowler (1986). Moreover the speech–action-repository (SAR) is assumed to be a neurofunctional model of non-symbolic (i.e., without semantics), supramodal (i.e., modality independent) syllable processing, which integrates higher-level (i.e., cortical) sensorimotor representations. In terms of speech processing, this syllable processing level is located between higher-level lexical processing (mental lexicon; cf. Levelt, 1992) and lower-level (i.e., subcortical) motor execution (cf. Riecker et al., 2005). The SAR model is based on simulation experiments (Kröger et al., 2009, 2011) that integrated an associative and self-organizing neural network approach (Kohonen, 2001) comprising two kinds of maps, i.e., a neural self-organizing map and neural state maps. Each of these maps comprises neurons, which represent different syllabic information (see Figure 1). Within the SAR model it is assumed that the syllabary is a supramodal hub linking motor and sensory (somatosensory and auditory) higher-level representations of frequent syllables (Kröger et al., 2011), which involves a brain network rather than one single region. In the current SAR approach, the syllabary not just stores a motor plan (gesture scores) for each frequent syllable. In addition an auditory representation (i.e., the subject knows what the syllable sounds like before he/she produces the syllable) and a somatosensory representation (i.e., the subject knows what the production of the syllable “feels” like) is stored. These representations are linked by a self-organizing supramodal map (phonetic map, Figure 1). Each model neuron within this neural map represents a specific phonetic^1^ realization of a frequent syllable and more than one phonetic realization of a syllable can be stored here. The sensorimotor knowledge is stored by synaptic link weights, i.e., neural mappings, between neurons of the phonetic map and neurons of the state maps, i.e., motor plan map, auditory map, and somatosensory map, hosting motor and sensory (somatosensory and auditory) representations of a syllable, if it is activated. The supramodal phonetic map is self-organizing and this map and its mappings toward the motor and sensory state maps are trained during speech acquisition (Kröger et al., 2009, 2011). Therefore, the phonetic map as well as the mappings toward motor and sensory maps can be interpreted as a part of long-term memory while the motor and sensory state maps are interpreted as parts of short-term memory (ibid.).

Due to the fact that the neural mappings between phonetic map and motor and sensory state maps comprise the main sensorimotor knowledge of frequent syllables it is assumed in our approach that the mapping between phonetic map and motor as well as between phonetic map and sensory maps is dense (i.e., a bulk of intersecting connections of model neurons, Figure 1).

Since motor representations occur in the frontal lobe while auditory and somatosensory representations occur in the temporal and parietal lobe (cf. Bohland and Guenther, 2006; Ghosh et al., 2008), it is hypothesized that there is a phonetic map which is anatomically implemented as a supramodal hub in order to allow integration of motor and sensory representations, i.e., state maps in terms of the SAR.

This assumption is examined in this fMRI study using a new reaction paradigm, which is based on simple syllables [consonant–vowel (CV) combinations] in homogeneous and heterogeneous blocks. Two complementary data analyses were performed. In a logical conjunction, activation for syllable processing independent of input modality and response mode was assessed, in order to support the assumption of existence of a *supramodal hub* (phonetic map) within a SAR. In addition priming effects in the BOLD response in homogeneous vs. heterogeneous blocks were measured, in order to detect brain regions, which indicate reduced activity during multiple production/perception repetitions of a specific syllable in order to determine *higher-level state maps* (motor plan, auditory, and somatosensory short-term memory state maps).

## Materials and methods

### Participants

In this pilot study, 20 data sets were recorded from five healthy male subjects who participated four times each. Participants were native speakers of German between 21 and 29 years old. Any health problems and medications that might affect cognitive function and brain activity, like neurologic or psychiatric diseases, were excluded. The handedness of the participants was tested with a German translation of the Edinburgh Handedness Inventory (Oldfield, 1971) to verify right handedness (Laterality Quotient ≥80). Non-verbal intelligence quotient (IQ) was tested with the short version of the Culture Fair Intelligence Test (CFT 20-R; Weiß, 2005). The participants were recruited from the local community. They were informed about the content of the experiment and risks of magnet resonance (MR). They consent in accordance with the guidelines established by the RWTH Aachen University and University Hospital Aachen. The experiment is approved by the University Hospital Aachen Ethics Board.

### Stimuli and procedure

**Figure 2:**
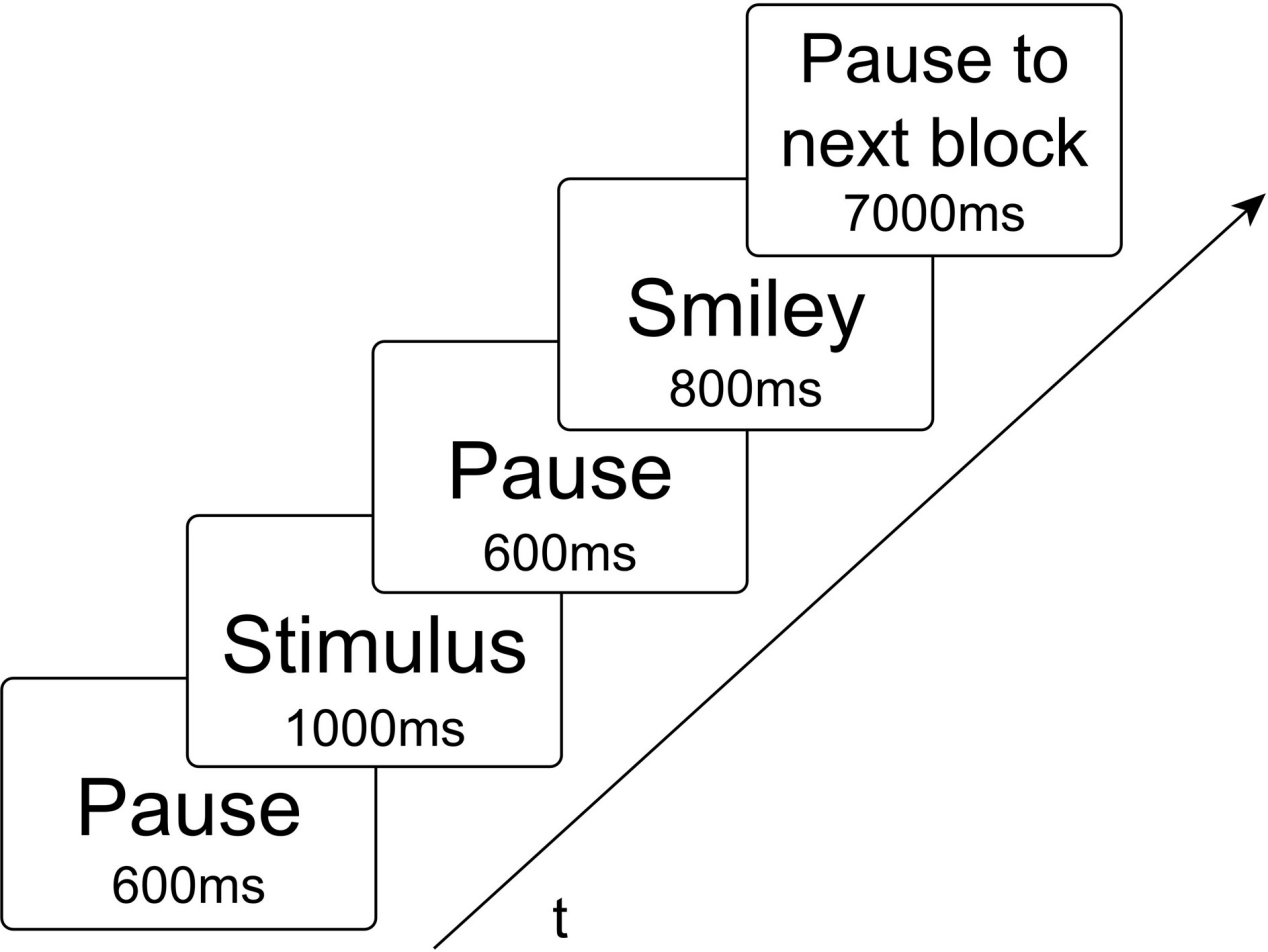
**Time-series of each stimulus presentation within a time of repetition of 3000 ms.** During presentation of the smiley no fMRI scans were made.

Experimental stimuli consisted of non-meaningful CV syllables, whereby C was represented by the voiced plosive [b] or the glottal stop [?] in combination with the vowels V = [a:], [e:], [i:], [o:], and [u:]. These syllables were acoustic records of a female speaker and visual characters implemented with the Software Presentation. Due to the experimental findings regarding the mental syllabary it was decided in this study to take only simple syllables. Thus, it is ensured that within this experiment only cortical representations related to the syllabary will be activated (Levelt and Wheeldon, 1994; Cholin et al., 2006). These stimuli were mixed into two different types of blocks. Homogeneous blocks consist of ten same CV syllables (exactly same token), containing each either CV syllables including [b] or CV syllables including [?]. Heterogeneous blocks consisted of five different syllables, which were randomly repeated two times in a block [e.g. bo-be-(pause)-bo-ba-be-ba-bi-bi-bu-bu]. These blocks either include CV-syllables with [b] or CV-syllables with [?]. A smiley appeared after each stimulus cueing the subject to respond now. There were ten different homogeneous blocks and two different heterogeneous blocks in each condition. The two heterogeneous blocks per condition were randomly chosen. Due to the duration of the blocks (see below) and in consequence, in order to ensure participants attention, awareness and physical condition it was decided to take only two heterogeneous blocks. Each of the blocks was repeated including a target [?E:] or [bE:] randomly presented in order to hold concentration. Totally there were 20 homogeneous blocks and 4 heterogeneous blocks randomly presented to the participants in each of four tasks. Each block lasted 40 s, including 10 stimuli [each presented 1000 ms; mean duration of auditory stimuli was 0.787 (0.094)], 10 smileys (each 800 ms), including pauses between stimulus and smiley as well as to the next stimulus (1200 ms), and if appropriate a target with smiley and pause (3 s), and further a 7 s pause to the following block (see Figure 2). The participants had to react with a button press when they see or hear a target. Blocks without a target included a 3 s pause randomly inserted in the block instead. The four tasks (conditions) differed with respect to (1) the presentation mode (visual vs. auditory), and (2) to the response mode (overt vs. covert). This resulted in a total of four task conditions (Table 1). The order of tasks was counterbalanced across participants. During one task the participants had to read aloud the syllables shown on a screen even when a smiley appears (READ). During another task they had to repeat the syllables presenting over headphones (REPEAT). The other two tasks were in the same presentation mode but the participants had to fulfill them in covert in place of overt speech (SILENT_READ AND SILENT_REPEAT). Each task lasted about 17 min. A sparse scanning procedure, where image acquisition pauses during smiley presentation, was used that allowed subjects to produce utterances in relative silence and avoids movement-related artifacts.

**Table 1:**
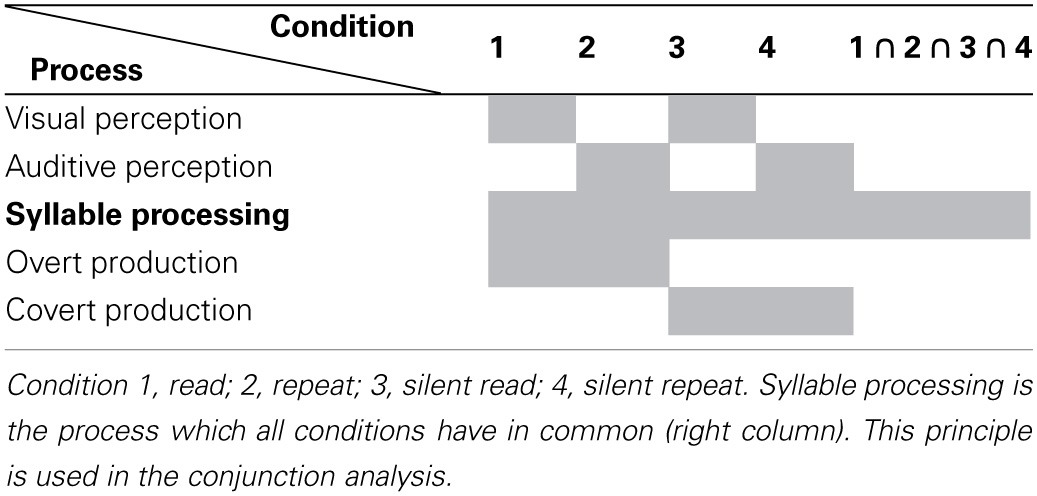
**Schematic representation of the processes taking place during the four different conditions**.

Condition 1, read; 2, repeat; 3, silent read; 4, silent repeat. Syllable processing is the process which all conditions have in common (right column). This principle is used in the conjunction analysis.

### Data acquisition and analysis

The experiment and data acquisition took place within a Siemens Magnetom Trio 3T Scanner. We obtained T2^*^ weighted functional images [time echo (*TE*) = 40 ms, time repetition (*TR*) = 3000 ms, flip angle = 90°, 39 slices, field of view (*FOV*) = 192 mm] using Echo Planar Imaging (EPI) acquisition. Each functional sequence consisted of thirty-nine 1.9 mm thick axial slices, positioned to image around the perisylvian fissure of the brain. A total of 1352 scans (4 × 338) were acquired for each subject. After the experiment we obtained a T1 weighted anatomical volume using magnetization-prepared rapid acquisition with gradient echo (MP-RAGE) sequence of about 9 min 50 s (*TE* = 3.03 ms, *TR* = 2300 ms, *FOV* = 256 mm, slice thickness 1 mm, 176 slices, flip angle = 9°).

Functional data preprocessing was conducted using SPM8 on Matlab 7.10 platform (MathWorks Inc., Natick, MA). Using standard methodology, data were adjusted for slice timing and motion corrected, spatially normalized to MNI space, and smoothed (8 mm FWHM Gauss Kernel) for each session.

A block-design analysis was conducted at the individual level. The statistical evaluation was based on a least-squares estimation using the general linear model for serially auto-correlated observations (Friston, 1994; Friston et al., 1995a,b; Worsley and Friston, 1995). To account for magnetic saturation effects, the first three scans of each time-series were discarded. Thus, 335 scans per task were admitted into the analyses. Because every subject fulfilled four different tasks, each during four sessions, a total of 5360 scans per subject were included in the analyses. The design matrix was generated with a synthetic haemodynamic response function (Josephs et al., 1997; Friston et al., 1998). The δ-functions of the stimulus onsets for each condition (READ, REPEAT, SILENT_READ, SILENT_REPEAT) were convolved with the canonical haemodynamic response with a distribution of 33 s (Friston et al., 1998). Each condition was contrasted against the implicit (resting) baseline, yielding the beta estimates for each condition in each participant.

To assess shared networks of syllable processing, independent of different input modalities and response modes, a conjunction analysis was performed. Inferences relating to consistency and generalizability of findings are reported using across-task and across-subject conjunctions of effects to identify common regional activity in each individual. The logical conjunction analysis was implemented to determine activation of syllable processing independent of input modality and response mode, representing supramodal syllable processing. This was implemented by calculating contrasts per condition per subject. A conjunction of these contrasts was computed per subject. Using the ImCalc tool of SPM8, these images were used to generate a common brain map comprising activated regions of all subjects at a level of *p* < 0.001 (uncorrected) to get overlapping areas according to the following formula: (*i*^1^ > 0) + (*i*^2^ > 0) + (*i*^3^ > 0) + (*i*^4^ > 0) + (*i*^5^ > 0). In each bracket it is defined that each conjunction per subject (*i*^1^ = subject 1, *i*^2^ = subject 2, *i*^3^,…) is saved in binary code. That means that each voxel satisfying the condition *p* < 0.001 (uncorrected) has value 1 and other voxels value 0. The values of the respective voxels in every participant's map are summed up. Within the resulting brain map overlapping regions are identified by a threshold of 2 (two subjects), 3 (three subjects), 4, or 5 and different colors^2^. We used the SPM8 Anatomy Toolbox to identify the cytoarchitectonic localization of the effects and to compare common regions of syllable processing activation within the group (Tables 2, 3).

**Table 2:** **Shared activated regions during supramodal syllable processing of at least 2 subjects**.

**Cluster size (voxels)**	**Local maximum in macroanatomical structure**	***x***	***y***	***z***	**No. of subjects**	**Percent of cluster volume in cyto-architectonic area**	
Cluster 1 (5328)	R Calcarine sulcus	10	−100	0	5	12.2	R Area 17
						11.2	L Area 17
						11.0	Area 18
Cluster 2 (227)	R PrCG^*^	54	−6	46	3	65.8	Area 6
Cluster 3 (80)	L PrCG^*^	−46	−4	44	3	80.8	Area 6
Cluster 4 (29)	L IFG^*^	−60	4	12	2	55.6	Area 44

References to cytoarchitectonic maps: area 6, Geyer (2003); areas 17/18, Amunts et al. (2000); area 44, Amunts et al. (1999). Cluster overlap with cytoarchitectonic areas is listed if it exceeds 10%. L, Left; R, Right; xyz, MNI coordinates; No., number of subjects ≥2;

* p < 0.001 in binomial test; PrCG, precentral gyrus; IFG, inferior frontal gyrus.

**Table 3:** **Shared activated regions during syllable priming of at least 2 subjects**.

**Cluster size (voxels)**	**Local maximum in macroanatomical structure**	***x***	***y***	***z***	**No. of subjects**	**Percent of cluster volume in cyto-architectonic area**	
Cluster 1 (1205)	L SPL^*^	−26	−76	46	4	22.1	SPL (7A)
						13.9	Area 2
						10.2	hIP3
Cluster 2 (1055)	R MTG^*^	58	−38	4	4		
Cluster 3 (695)	R Insula^*^	36	30	0	3	12.4	Area 45
Cluster 4 (642)	L IFG^*^	−52	10	28	4	37.9	Area 44
						15.8	Area 45
Cluster 5 (468)	L Temporal pole^*^	−54	10	−8	3		
Cluster 6 (407)	L MTG^*^	−62	−24	−2	3		
Cluster 7 (349)	L SMA^*^	−4	6	54	4	38.8	L Area 6
						14.4	R Area 6
Cluster 8 (72)	L SMG^*^	−58	−44	24	2	76.6	IPC (PF)
						11.3	IPC (PFm)
Cluster 9 (56)	R Precuneus^*^	8	−66	38	2	37.3	SPL (7A)
						25.7	SPL (7M)
						17.6	SPL (7P)
Cluster 10 (56)	L IFG	−44	32	24	3	28.8	Area 45
Cluster 11 (39)	R IFG	58	14	32	2	44.2	Area 44
						13.5	Area 45
Cluster 12 (37)	L IFG	−52	8	6	2	78.7	Area 44

References to cytoarchitectonic maps: area 2, Grefkes et al. (2001); areas hIP3/7A/7M/7P, Scheperjans et al. (2008); area 6, Geyer (2003); areas 44/45, Amunts et al. (1999); areas PFm/PF, Caspers et al. (2006). Cluster overlap with cytoarchitectonic areas is listed if it exceeds 10%. L, Left; R, Right; xyz, MNI coordinates; No., number of subjects ≥2;

* p < 0.05 in binomial test; SPL, superior parietal lobe; MTG, middle temporal gyrus; IFG, inferior frontal gyrus; SMA, supplementary motor area; SMG, supramarginal gyrus; IPC, inferior parietal gyrus.

Further the main syllable priming effect in the BOLD response in homogeneous vs. heterogeneous blocks, i.e., syllable priming, was calculated, reflecting the reduced effort of accessing a syllable representation. Therefore, one contrast per subject was computed, considering the distinction of heterogeneous greater than homogeneous blocks, i.e., syllables priming. The main effect image per subject was saved as binary cluster image and, even like described before, calculated in ImCalc to get common regions of activation including all subjects at a level of *p* < 0.001 (uncorrected).

In addition, in order to provide an additional measure of the stability and reliability of the internal data structure underlying these results, we ran binomial tests over the contrast images of each task (READ, SILENT_READ, REPEAT, SILENT_REPEAT) for each scanning session (1–4) of each subject (1–5), giving a total of 79 values for each local maximum observed in the conjunction analysis (subject 1 did not complete all four tasks in the first scanning session, thus there are 79, not 80, data points). For the binomial tests, the data were binarised, i.e., assigned the value 1 if there was a positive effect for this voxel in this subject × task × scanning session combination, and 0 if the effect was smaller or equal to zero. The binomial test then assessed the statistical probability of an equal distribution of values 1 and 0. Under the null hypothesis, this probability was 50%. A comparable analysis was run cluster-wise for the HET > HOM priming effects.

## Results

All neuroanatomical abbreviations can be found in Table A1 of the Appendix.

### Supramodal syllable processing

**Figure 3:**
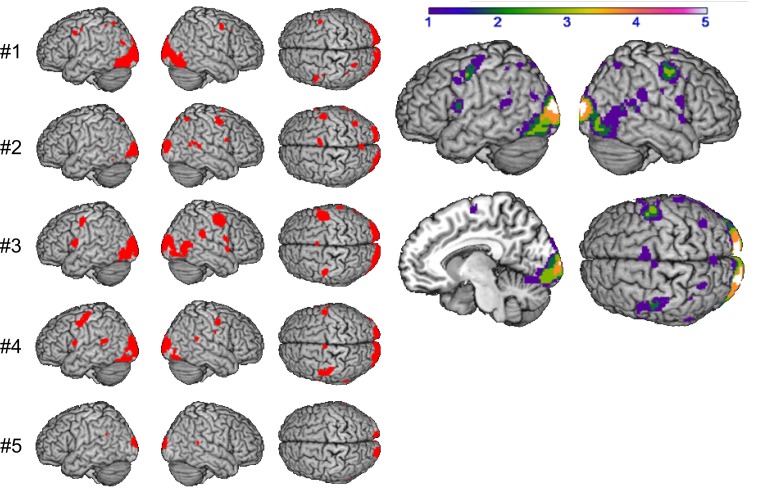
**Supramodal syllable processing: conjunction analysis per subject #1–#5 (left) and in group (right): shared of 1 (purple) to 5 (white) subjects (*p* < 0.001 uncorrected)**.

The logical conjunction analysis assessing activation for syllable processing independent from input modality (auditory, visual) and response mode (overt, covert) calculated with the four contrasts (READ, SILENT_READ, REPEAT, SILENT_REPEAT) revealed supramodal syllable processing, individually and comparable over subjects in frontal brain regions (see Figure 3). By computing overlapping areas of all subjects using the ImCalc tool of SPM8, this resulted in a shared activation network of syllable processing of one (purple) to five subjects (white) (*p* < 0.001, uncorrected). This network comprises frontal areas, i.e., bilateral precentral gyrus (PrCG, area 6) and left inferior frontal gyrus (IFG, area 44) as well as occipital areas, i.e., visual cortex (area 17) (see Figure 3 and Table 2).

### Syllable priming

**Figure 4:**
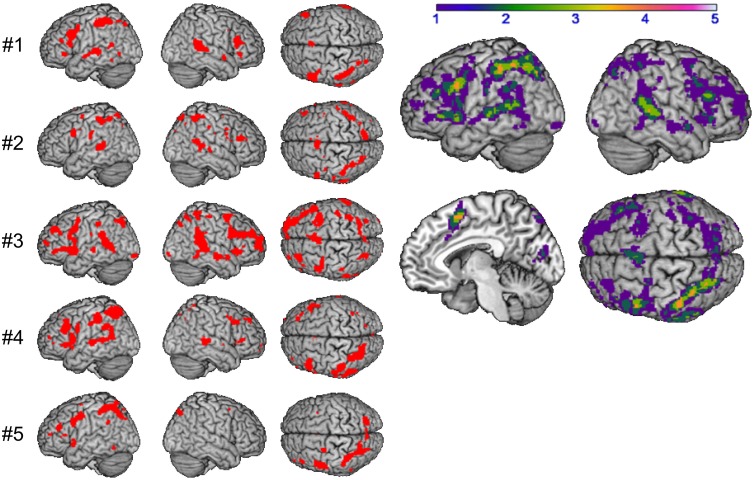
**Main effect of syllable priming per subject #1–#5 (left) and in group (right): shared of 1 (purple) to 4 (orange) subjects (*p* < 0.001 uncorrected)**.

A computation of the main effect of heterogeneous vs. homogeneous blocks was implemented to determine priming effects in the BOLD response, reflecting the reduced or increased effort of accessing the syllable representation for each subject. The resulting conjunction images were compared by using the ImCalc tool. The homogeneity priming revealed an activation pattern, comprising frontal areas, i.e., bilateral IFG (area 44), left supplementary motor area (SMA), right insula, temporal areas, i.e., temporal pole and bilateral middle temporal gyrus (MTG), and parietal areas, i.e., bilateral superior parietal lobe (SPL) and left supramarginal gyrus (SMG, see Figure 4 and Table 3). Activation within these areas was usually more pronounced in the left hemisphere, with overlap of at least three subjects.

### Binomial tests for task effects over subjects and sessions

The binomial test assessing the statistical probability of an equal distribution of values “1” and “0” revealed that the empirical distributions differed significantly from an equal (i.e., random) distribution, with significance levels of *p* = 0.001 for each region (right precentral gyrus (PrCG), left PrCG, and left IFG).

### Binomial tests for syllable priming

Similarly, for syllable priming, the binomial test showed results largely comparable to those of the standard GLM conjunction analysis reported above. Except for parts of right and left IFG (see Table 3), all other regions showed distributions differing significantly from the null hypothesis (i.e., equal distribution) at *p* = 0.05.

## Discussion

### Summary and interpretation

The aim of the current study was to investigate possible cortical locations of the SAR model of Kröger et al. (2009, 2011) in order to support the assumption of existence of a *supramodal hub* (phonetic map) which is assumed to be anatomically implemented in order to associate representations of *higher-level state maps* (motor plan, auditory, and somatosensory short-term memory state maps). This was examined in two distinctive analyses: (1) by controlling different input modalities and response modes in order to get supramodal syllable processing, and (2) by evoking syllable priming effects in order to determine activated regions during access to sensorimotor representations (state maps) in terms of the SAR.

**Figure 5:**
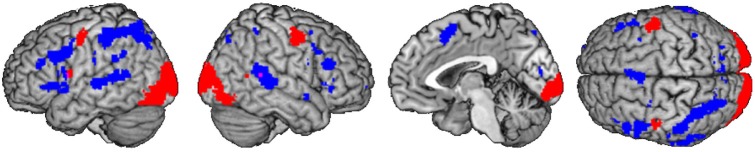
**Comparison of shared activation networks: supramodal syllable processing (red) with main effect of syllable priming (blue) (*p* < 0.001 uncorrected) shared of subjects ≥2**.

The analysis of supramodal syllable processing resulted in a significant activation network, involving frontal areas, i.e., bilateral PrCG as well as left IFG (area 44, Figure 3). In the framework of the present study, these regions are related to the phonetic map as a supramodal hub. Furthermore, syllable priming evoked activation in frontal areas, i.e., bilateral IFG (area 44), left SMA, and right insula as well as in temporal areas, i.e., left temporal pole and bilateral MTG as well as in parietal areas, i.e., bilateral SPL and left SMG (Figure 4). This neurofunctional network represents access to different modality specific representations (state maps). Figure 5 summarizes activated areas representing the SAR, i.e., *supramodal hub* (red) as well as *higher-level state maps* (blue). These findings are consistent with the notion of a SAR (Kröger et al., 2009, 2011).

Within this study parts of frontal, temporal, and parietal areas were found to be activated during syllable processing. Frontal regions [IFG (area 44), bilateral PrCG and left SMA] represent preparative aspects of syllable processing (e.g., Riecker et al., 2005; Bohland and Guenther, 2006; Brendel et al., 2011). These areas as well as the superior cerebellum are activated during speech motor planning (e.g., Riecker et al., 2005; Bohland and Guenther, 2006; Ghosh et al., 2008). This is in line with the current findings. In a study, which controlled syllable frequency activation, Papoutsi et al. (2009) found activation in the PrCG as well as IFG bilaterally during production of low-frequent syllables. In the current study, among others, the same regions were found. Moreover, Riecker et al. (2005) as well as Eickhoff et al. (2009) found the IFG (area 44) as starting point of speech initiation. Previous studies provide further evidence of the PrCG and IFG (area 44) to be important during syllable preparation and provide evidence for these regions to play a major role in the SAR. In the framework of the present study PrCG and IFG (area 44) might relate to the supramodal hub on the one hand, and IFG (area 44/45) and SMA to the motor plan state map of the SAR on the other hand.

It is important to note that activations of the PrCG and IFG (area 44/45) during supramodal syllable processing and during syllable priming did not overlap (see Figure 4). This supports the assumption of different areas to represent different kinds of maps within the SAR, i.e., the supramodal hub and the state maps. However, further investigations have to confirm these new findings.

In the temporal lobe bilateral activation of the MTG was found. We assume the activation of this area to represent access to the auditory state map of the SAR. In previous literature the MTG is described in connection with lexical and semantic access (Hickok and Poeppel, 2007), but in the current fMRI investigation stimuli were meaningless. Rimol et al. (2005) determined that the MTG plays a role during phonetic encoding of syllables and Chang et al. (2008) reported children who stutter had less gray matter volume in the bilateral MTG relative to fluently speaking children. This might support the role of the MTG in accessing the auditory state map of high-frequent syllables within the SAR. But further investigations are needed to explain the role of the MTG more precisely.

Syllable priming effects were found in the left SMG as well as bilateral SPL. In the framework of the present study, these activations might represent access to the somatosensory state map of the SAR. This is supported by different fMRI studies in which somatosensory syllable processing was found to take place in the ventral somatosensory cortex and anterior SMG (Hashimoto and Sakai, 2003; Ghosh et al., 2008; Tourville et al., 2008). However, parietal areas were also associated with verbal working memory (Smith et al., 1998) or a phonological store, which can be temporarily activated by incoming verbal information (Jonides et al., 1998). Henson et al. (2000) assumed that SPL and SMG participate in phonological recoding of visually presented verbal materials. It cannot be ruled out completely that some aspects of activation of SPL and/or SMG relate to phonological processes within the current study. Furthermore, the posterior parietal cortex is traditionally associated with attention (Posner and Petersen, 1990); therefore, priming effects in the parietal lobe could partly reflect attention as a cognitive function in the current study as well.

Activation of the visual cortex during all conditions (supramodal syllable processing) is due to the fact that a smiley is presented during every condition cueing the subject to speak. Because this region is not sensitive to the syllable priming effect it is not further interpreted to be relevant to the SAR.

Furthermore, bilateral activation was found in premotor cortices. In order to examine whether activations on the right hemisphere are due to the button press, which was performed with the left hand after a target appeared, we conducted a control analysis, comparing data including target responses to data including no target responses. Except for the fact that blocks with targets were analysed separately from those without targets, this analysis was identical to the original analysis. This comparison revealed a right hemispheric involvement also during syllable processing when no buttons were pressed. Thus, the right premotor activation seems to be independent of button press activation, but truly related to syllable processing.

## Limitations

Within this study design it could not be analyzed in greater detail, if temporal regions represent auditory, parietal regions represent somatosensory, and frontal regions represent only motor plan functions. To evaluate each state map within the SAR another study design with tasks that can be differentiated clearer has to be generated. Furthermore, using exactly the same tokens to represent auditory stimuli in the homogeneous blocks could result in facilitation of acoustic information processing besides syllable processing. However, this is likewise true for, and in part due to, the processing of the visual stimuli, which were also identical. Thus, whereas auditory (and likewise) facilitation may indeed contribute to the priming effect, these are rather complementary and thus unlikely to drive the supramodal effects reported here.

Within the approach of the SAR it is described that the supramodal hub and the state maps are simultaneously activated (Kröger et al., 2009). With aid of our analyses we cannot determine whether activation of supramodal syllable processing and syllable priming within the cortical regions is temporally simultaneously or temporally successively. Repeating this experiment in further subjects using simultaneous dynamic casual modeling in addition, the order of activation and the direction of activation might be determined. This will be examined in a larger group of participants.

Two different kinds of blocks were used in this study, i.e., 10 homogeneous and 2 heterogeneous blocks. In fact, if having 2 instead of 10 heterogeneous blocks induced some bias in the data, this bias would work against the hypothesis that there is syllable priming, not in favor of it. This is because of the potentially higher amount of variability in the relatively small number of blocks. Nonetheless, the differences of the beta estimates were consistently higher than 0, i.e., providing reliable effects—even across subjects.

Given that the group of participants was small (*n* = 5), even though the data set itself was larger by virtue of the repeated scans and multiple tasks, further of a supramodal hub and its mappings to the sensorimotor state maps in a larger sample are desirable.

## Conclusion

The current study was to the best of our knowledge the first to investigate the assumption of a *supramodal hub* and different *sensorimotor representations* (state maps) in two different analyses: (1) by controlling different input modalities and response modes and (2) by evoking syllable priming. This investigation revealed new insights in syllable processing in terms of a SAR. The cortical regions, which were found in this study, are in line with the SAR approach by Kröger et al. (2009, 2011). In order to provide more evidence for this, there will be further syllable processing investigations.

### Conflict of interest statement

The authors declare that the research was conducted in the absence of any commercial or financial relationships that could be construed as a potential conflict of interest.

## Footnotes

^1^(1) Within this approach “phonetic” does not just cover “auditory” but as well “motor” and “somatosenory.” That means, the term “phonetic” covers all sensory domains, which are important for speech and in addition the motor domain (including articulation), because speech is generated basically by vocal organ movements and then transferred into an acoustic speech signal at the level of the vocal tract. (2) “Phonetic” is used as dissociation of the term “phonological”: the phonetic map/hub is the central layer of the mental syllabary, while a phonological hub might be a central layer of the mental lexicon. The term “phonetic” underpins, that in this approach, storage of syllables (the mental syllabary), consisting of motor and sensory information without meaning, is meant. (3) “Phonetic” means furthermore “comprising every speech modality,” i.e., “supramodal” or “amodal”: “phonetic” comprises motor, auditory, and somatosensory information and the hub is meant as neural storage entity, which processes information of these three different modalities.

^2^A conjunction analysis of READ, SILENT_READ, REPEAT, and SILENT_REPEAT that was significant for an individual participant at *p* < 0.001 effectively means that, for a voxel surviving this analysis, each single contrast was significant at *p* < 0.001, so the effective *p*-value for this voxel is *p* = (0.001)^4^ = 0.000000000001. This, in turn, means that a voxel which is shared by more than one subject has an effective *p*-value of *p* = (0.000000000001)^*i*^, with *i* being the number of subjects for which the maps overlay at this point.

## Appendix

**Table A1:** **List of cortical abbreviations divided into cortical lobes**.

**Lobe**	**Abbreviation**	**Term**
Frontal	IFG	Inferior frontal gyrus
	SMA	Supplementary motor area
	PrCG	Precentral gyrus
Temporal	MTG	Middle temporal gyrus
Parietal	SMG	Supramarginal gyrus
	IPC	Inferior parietal cortex
	SPL	Superior parietal lobe
